# Effect of Transcatheter Intraarterial Therapies on the Distribution of Doxorubicin in Liver Cancer in a Rabbit Model

**DOI:** 10.1371/journal.pone.0076388

**Published:** 2013-10-08

**Authors:** Bin Liang, Fu Xiong, Hanping Wu, Yong Wang, Xiangjun Dong, Shaofeng Cheng, Gansheng Feng, Guofeng Zhou, Bin Xiong, Huimin Liang, Xiangwen Xia, Chuansheng Zheng

**Affiliations:** 1 Department of Radiology, Union Hospital, Tongji Medical College, Huazhong University of Science and Technology, Wuhan, China; 2 Department of Radiology, University Hospitals Case Medical Center, Case Western Reserve University, Cleveland, Ohio, United States of America; Northwestern University Feinberg School of Medicine, United States of America

## Abstract

**Background and Aims:**

Transcatheter intraarterial techniques can effectively deliver chemotherapeutic agents to tumor and improve the efficacy of chemotherapy. The present study is designed to evaluate the effect of transcatheter intraarterial techniques on the distribution of doxorubicin in relation to blood vessels in liver cancer.

**Methods:**

VX2 tumors were implanted in the livers of 32 rabbits. The animals were divided into 4 groups of 8 animals each. Group 1 (doxo iv) animals received doxorubicin intravenous injection; group 2 (doxo ia) received doxorubicin hepatic intraarterial infusion; group 3 (doxo ia + E) received doxorubicin hepatic intraarterial infusion followed by embolization; group 4 (doxo + L ia + E) received hepatic intraarterial infusion of doxorubicin mixed with Lipiodol followed by embolization. Ten minutes or 4 hours after treatment, the animals were sacrificed and tumors were sampled. Immunofluorescence techniques were used to evaluate the distribution of doxorubicin in relation to blood vessels.

**Results:**

Doxorubicin fluorescence was distributed around tumor blood vessels and decreased with distance from the blood vessels. Tumor cells in avascular and adjacent regions were not exposed to detectable concentrations of doxorubicin. Tumors in the group 2, 3 and 4 had a significant increase in doxorubicin penetration compared with the group 1 tumors (*P*<0.05). Among the three groups of transcatheter therapies, doxorubicin penetration distance in group 3 was significantly larger than that in group 2 and 4 (*P*<0.05), and no significant difference was found between group 2 and 4 tumors (*P*>0.05) at 10 minutes. In contrast, at 4 hours and in total, both group 3 and 4 tumors had significant increases in drug penetration compared with group 2 (*P*<0.05), and no significant difference was noted between group 3 and 4 tumors (*P*>0.05).

**Conclusion:**

Transcatheter intraarterial therapies improve doxorubicin penetration in liver cancer; nevertheless their effect on drug distribution is somewhat limited.

## Introduction

Liver cancer remains a substantial health challenge worldwide. Although resection, liver transplantation and ablation are curative therapies, only a small minority of patients are candidates for these treatments [1]. Transcatheter intraarterial therapies are widely used palliative treatments for unresectable primary and metastatic liver cancer [2] and have exhibited encouraging results in terms of survival [3], [4]. The rationale for intraarterial therapies stems from the observation that the blood supply of advanced liver cancers is mostly dependent on the hepatic artery [5]. Hepatic artery chemotherapeutic infusion, defined as injection of chemotherapy with or without lipiodol in the hepatic artery via selective catheter placement, is an important component of transcatheter therapies for liver cancer [6]. Although liver cancer is resistant to systemic chemotherapy, the tumor has proved to respond to some extent to hepatic artery chemotherapeutic infusion. Injection of chemotherapeutic drugs causes cytotoxic effect [7], and when combined with the following embolization of tumor-feeding vessel, i.e., transcatheter arterial chemoembolization (TACE), results in substantial tumor necrosis [8]. The enhanced sensitivity of liver cancer to chemotherapy is presumedly attributed to the effective delivery of highly concentrated dosed of chemotherapeutic drugs to the tumor.

However, the efficacy of chemotherapy on liver cancer remains controversial. Recent studies have shown that chemotherapy without embolization seemed to achieve a lower percentage of tumor necrosis compared with TACE [9], and that the addition of chemotherapy to embolization did not result in better effectiveness than embolization alone [10]. It is well known that to maximize the cytotoxic effects chemotherapeutic agents must penetrate deep to reach all cancer cells at a therapeutically effective concentration. Previous data suggested that when given subcutaneously or intravenously anticancer drug formed gradients within tumor, with high concentrations in the periphery and low concentrations in the center of the tumor [11], [12]. Although the drug concentration in tumor tissue has been shown to be much higher than that in the normal liver parenchyma [13], [14], anticancer drug delivered through an intraarterial route was located mainly around the embolized vessels in liver cancers undergoing TACE [15], [16]. The limited distribution of anticancer drug in tumor may account for the impaired efficacy of chemotherapy. An examination of drug distribution in liver cancer after transcatheter intraarterial therapies could help understand the therapeutic effect of chemotherapeutic infusion and develop strategies to modify drug distribution.

Doxorubicin is the most widely used single chemotherapeutic agent in the transcatheter therapy for liver cancer. Infusion of the drug exhibited favorable response rate and survival in selected candidates [17]. In addition, the drug is amenable to direct detection by fluorescence microscopy because of its fluorescent property. Doxorubicin has been used to study drug distribution in relation to tumor blood vessels, recognized by the endothelial cell marker CD31, in animal and human tumors [11], [12]. In the present study, we utilized the fluorescence of doxorubicin to study the effect of transcatheter techniques on drug distribution in a VX2 tumor model of liver cancer.

## Materials and Methods

### Experimental design

A total of 32 adult New Zealand White rabbits weighing 2.5–3.0 kg were used in this study. VX2 tumors were inoculated in the livers of the animals. Seventeen days after tumor inoculation, each animal underwent magnetic resonance (MR) imaging to detect tumor formation. One day after MR imaging, the tumor-bearing rabbits were randomly assigned into four groups of 8 animals each and were treated. In group 1 (doxo iv), animals received doxorubicin intravenous injection. In group 2 (doxo ia), animals underwent doxorubicin hepatic intraarterial infusion. In group 3 (doxo ia + E), animals were subjected to the hepatic intraarterial infusion of doxorubicin followed by embolization with vascular occlusion agent. In group 4 (doxo + L ia + E), animals received hepatic intraarterial infusion of doxorubicin mixed with lipiodol followed by the embolization. Ten minutes or 4 hours after interventional treatment, four animals in each group were sacrificed, respectively, and tumor samples were harvested for immunofluorescence.

### Tumor inoculation

Animal experiments were conducted according to the Guide for the Care and Use of Laboratory Animals of Huazhong University of Science and Technology, as approved by the Animal Care Committee of Hubei Province, China. The animals were anesthetized with an intravenous injection of sodium pentobarbital (30 mg/kg body weight), and all efforts were made to minimize suffering. VX2 tumor is a highly malignant carcinoma derived from a virus-induced papilloma of rabbits. The tumor can be grown in the liver of rabbit and show some biological similarities to human hepatocellular carcinoma, which has become an optimal animal liver tumor model for the development of catheter-directed therapies [18]. The VX2 strain was maintained by successive transplantation into the hind limb of carrier rabbits. For the tumor inoculation, the tumor was first harvested from the carrier rabbit and minced into 1-mm^3^ in Hanks' solution. Then, the liver of the recipient rabbit was exposed through a subxiphoid abdominal incision, and the tumor fragments were embedded 10-mm deep into the left liver lobe. A small piece of gelatin sponge was placed into the wound to obtain hemostasis and the abdominal wall was closed thereafter.

### MR imaging

MR imaging was performed by using a 1.5-T unit (Magnetom Avanto; Siemens Medical Solutions, Erlangen, Germany). Rabbits were imaged in the supine position using the combination of a six-channel body phased-array coil and a two-channel spine phased-array coil. All animals underwent T2-weighted turbo spin-echo sequence with the following imaging parameters: TR/TE = 3700/87 ms, 4-mm slice thickness, 15% intersection gap, 168-Hz/pixel BW, 200×200 mm^2^ field of view, 320×320 matrix, turbo factor  = 11, averages = 2.

### Transcatheter techniques

Transcatheter procedure was performed in the animals under the guidance of digital subtraction angiography (Altis Zee Celling, Siemens Medical Solutions, Muenchen, Germany). Briefly, the right femoral artery was accessed through open puncturation with an 18-gauge needle and catheterized with a 4-F vascular sheath (Terumo, Tokyo, Japan). A 4-F Cobra visceral catheter (Terumo) was first used to select the celiac axis, and then a 2.7-F coaxial catheter system (Terumo) was advanced coaxially through the 4-F catheter to the proper hepatic. After the inoculated tumor was confirmed to receive its blood supply from the hepatic artery with digital subtraction angiography of the proper hepatic artery, subsequent treatments were carried out. Doxorubicin was given intraarterially or intravenously at a dose of 8 mg/kg to facilitate detection and quantification of drug auto-fluorescence. For material preparation, 8-mg/kg doxorubicin powder was dissolved in 1-mL saline water or mixed with 0.4-mL Lipiodol, and PVA particles 150–250 μm in size (PVA, Cook, Bloomington, USA), packaged as a vial of dry particles, were reconstituted with 10 mL contrast media. Animals in group 2 received the hepatic intraarterial infusion of doxorubicin solution. Group 3 animals received the doxorubicin solution infusion followed by embolization with 0.2-0.4 mL PVA mixture. Group 4 animals received infusion of the doxorubicin/Lipiodol mixture, followed by the PVA embolization. The embolization endpoint for groups 3 and 4 was the complete stasis of antegrade blood flow. For group 1 animals, doxorubicin solution was injected via ear vein without interventional procedure.

### Immunofluorescence and image analysis

Animals were humanly sacrificed 10 minutes or 4 hours after doxorubicin administration with intravenous administration of overdose sodium pentobarbital (100 mg/kg body weight). The selection of the two sacrifice time points was based on the previous observation that doxorubicin can be effectively transported to tumor cells through blood vessels in a 10-minute cycle time when used alone [19] and that, when mixed with Lipiodol, all doxorubicin can elute from the Lipiodol emulsion in less than 4 hours [20]. The samples that included the greatest diameter of tumor were harvested and then embedded immediately in optimum cutting temperature compound and frozen in liquid nitrogen. Cryostat sections 10 μm thick were cut at the greatest diameter of tumor, mounted on glass slides, and allowed to air-dry.

Doxorubicin auto-fluorescence was detected using Nikon TE2000 inverted fluorescence microscopy 465 to 495 nm excitation and 510 to 555 nm emission filters, and images of tissue section were captured with a Nikon DS-U3 camera. Blood vessels were recognized by the expression of CD31 membrane protein on endothelial cells. Following doxorubicin imaging, tissue sections were fixed in paraformaldehyde for 10 min, washed in PBS, and blocked with fetal calf serum (dilution, 1∶20; Roche) for 20 min to prevent non-specific antibody binding. Specimens were incubated overnight at 4°C with mouse anti-human CD31 monoclonal antibodies (dilution, 1∶20; Dako), then washed in PBS and stained with goat anti-mouse secondary antibody Fitc (dilution, 1∶50; Jakson) for 1 h at room temperature, after that stained with DAPI nuclear dye for 10 min. Finally, sections were cover-slipped with a mounting medium containing anti-fluorescein quencher. Anti-CD31 fluorescence representing endothelial cell was detected with 510 to 560 nm excitation and 590 nm emission filters, and DAPI-stained nuclear DNA localization was detected with 330–380 nm excitation and 420 nm emission filters.

Composite images of doxorubicin and CD31 fluorescence were generated using Image Pro Plus software. For CD31 images, Microvessel density (MCD) was evaluated using the counting method introduced by Weidner et al [21]. The tumor sections were firstly scanned at low magnification field (×40 magnification, 6.82 mm^2^) to find the areas that showed the most intense vascularization and then individual microvessels were counted in three different high magnification fields (×200 magnification, 0.27 mm^2^). The final MVD was the mean value of the counts of the three fields. For the evaluation of doxorubicin, three different high magnification fields (×100 magnification, 1.09 mm^2^) with high doxorubicin fluorescence were selected. Areas of necrosis and staining artifact were excluded.

The penetration of doxorubicin was measured using the Image Pro Plus software application and customized algorithms. Briefly, a doxorubicin image and a separate blood vessel image of the same field were firstly captured. In order to make the blood vessel image the same size as the doxorubicin image, an extra copy of the doxorubicin image was made, and was overlaid on top of the blood vessel image. The blood vessel image was then masked, so that all the blood vessels can be identified and be converted to white color with an intensity of 255. The remaining pixels in that image had an intensity of 0. This was called a binarized image. Any pixel that was not 0 was regarded as a blood vessel. The image then was converted and a new image was formed by using the function of distance filter. In this picture, the gray value of each point represented the distance of the point to the nearest vessel. The doxorubicin image was also switched to the binarized image in which each doxorubicin positive spot was represented as its center point. By using the Minimum function of software, the distance of each doxorubicin spot from the nearest blood vessel was measured. The data were tabulated to average out the doxorubicin penetration distance of the field. The mean penetration distance of the three fields was taken as the final doxorubicin penetration distance for each animal [22]. The count of doxorubicin-specific fluorescent spot in each field was simultaneously counted.

### Statistical analysis

Data are summarized as the mean ± standard deviation (SD). Differences between the groups were compared using one-way analysis of variance followed by LSD-*t* test. *P*<0.05 was considered statistically significant.

## Results

### VX2 tumor and transcatheter procedure

**Figure 1:**
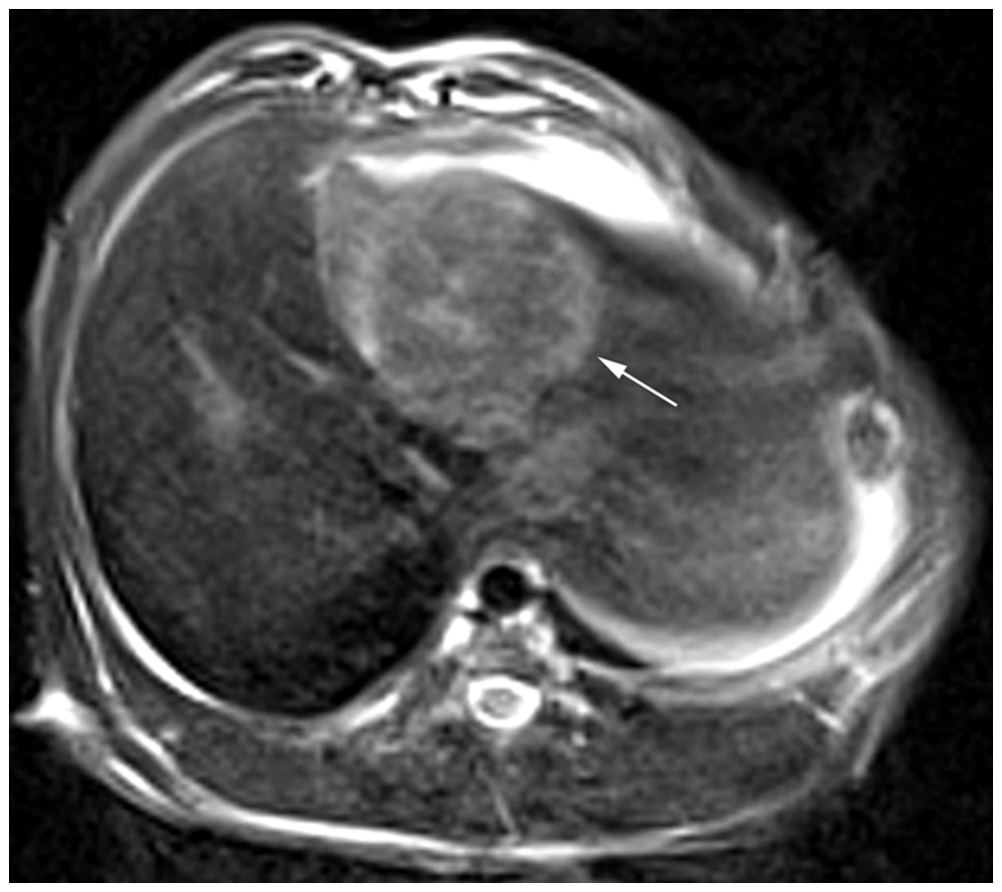
MR image of a VX2 tumor. T2-weighted MR image shows a hyper-intensity VX2 tumor in the left liver lobe (arrow).

**Figure 2:**
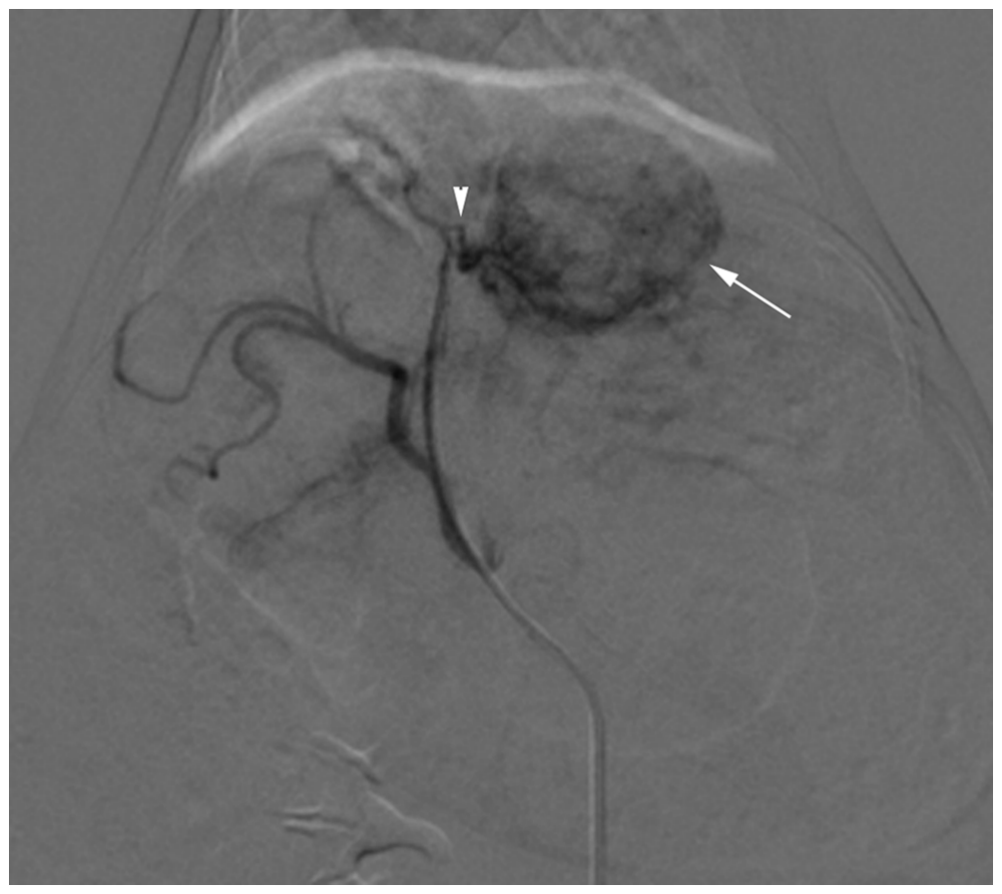
DSA image of a VX2 tumor. Selective left hepatic artery angiogram shows a hypervascular tumor (arrow) receiving its blood supply from the left hepatic artery (arrowhead).

VX2 tumor was successfully grown in the left liver lobe of each rabbit (Figure 1). Tumors ranged from 1.16–2.12 cm in diameter. The mean diameters of group 1, 2, 3, and 4 were 1.53 cm±0.27, 1.58 cm±0.30, 1.63 cm±0.25, and 1.49 cm±0.20, respectively, without significant difference between groups (*P* = 0.679). Transcatheter procedures were performed successfully in all animals (Figure 2).

### Microvessel density

**Figure 3:**
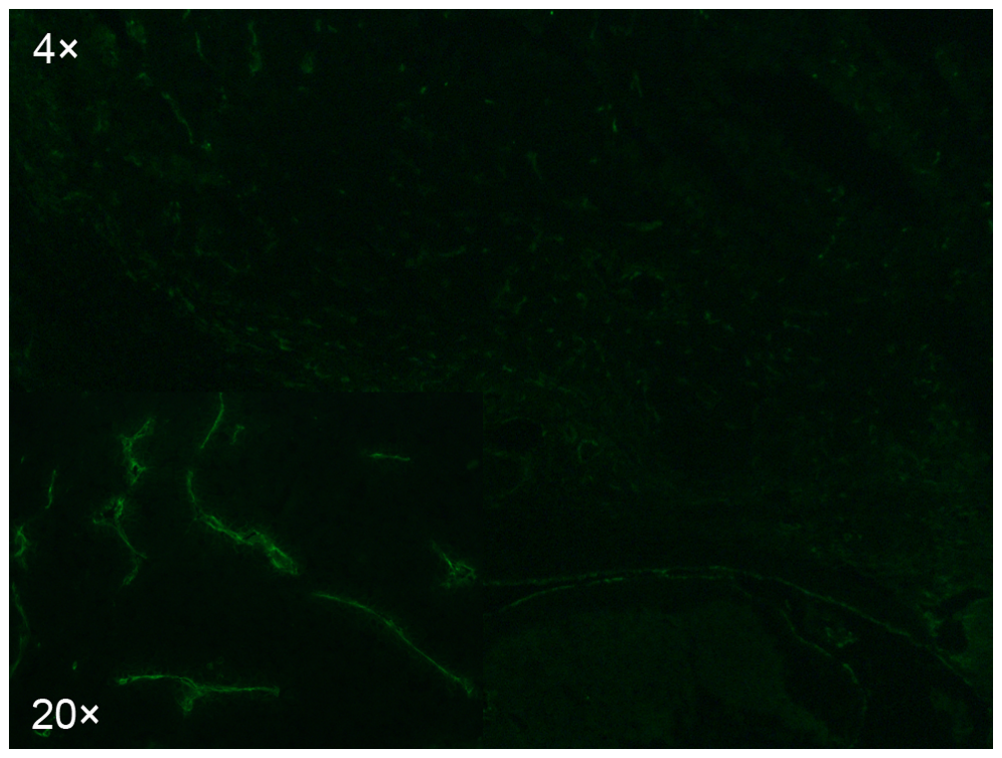
Immunofluorescence image of anti-CD31 stain. Photomicrograph of a representative VX2 tumor shows numerous blood vessels (green, recognized by CD31) at the edge of the tumor.

In the cryostat sections, microvessels were demonstrated as green fluorescence of separated single endothelial cell or connected cell cluster (Figure 3). Microvessels were heterogeneously distributed within the VX2 tumor, and the most intense vascularization was observed at the edge of the tumor. There was no significant difference in MVD between the four groups (*P* = 0.543) (Table 1).

**Table 1:** Microvessel density, doxorubicin penetration distance and count of doxorubicin fluorescence spot in four groups.

Groups	Time points	Microvessel density	Doxorubicin penetration distance (μm)	Count of doxorubicin fluorescence spot
**1 (n = 8)**	10 minutes	12.67±2.35	12.14±4.07	235.75±158.45
	4 hours	14.41±0.84	7.36±2.37	75.00±71.89
	Total	13.54±1.88	9.75±4.01	155.38±142.68
**2 (n = 8)**	10 minutes	12.83±1.04	40.54±7.23	2070.50±586.39
	4 hours	12.43±1.98	32.68±8.73	1137.00±588.12
	Total	13.50±1.76	36.61±8.53	1603.75±737.96
**3 (n = 8)**	10 minutes	14.17±2.44	76.29±31.29	3130.75±341.27
	4 hours	15.73±2.74	72.37±21.68	3190.00±647.95
	Total	14.95±2.54	74.33±25.01	3160.38±480.49
**4 (n = 8)**	10 minutes	15.34±2.65	38.21±7.19	1912.00±311.10
	4 hours	13.11±2.58	81.16±21.95	3681.50±367.31
	Total	14.22±2.69	59.68±27.49	2796.75±996.95

### Doxorubicin distribution and its relationship with microvessels

**Figure 4:**
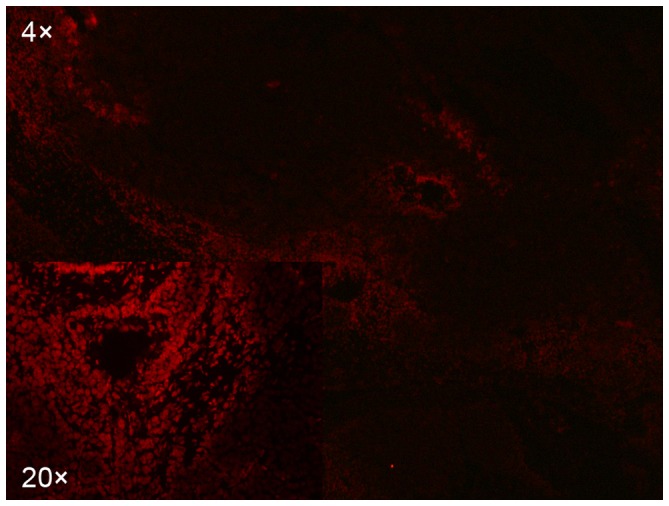
Immunofluorescence image of doxorubicin. Photomicrograph of a representative VX2 tumor shows doxorubicin auto-fluorescence (red) at the edge of the tumor.

**Figure 5:**
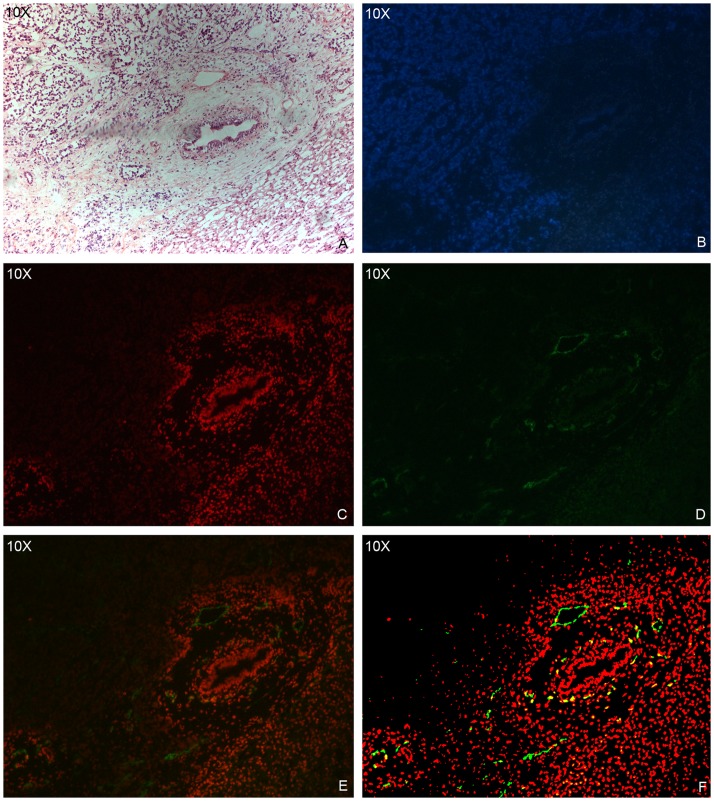
Histology images of a VX2 tumor. Hematoxylin-eosin (A), immunofluorescence (B, nucleus; C, doxorubicin; D, blood vessels) and composite (E, original composite; F, modified composite) images show the distribution of doxorubicin (red) in relation to tumor blood vessels (green).

Doxorubicin fluoresced red in the tumor sections. The drug-specific fluorescence was detected primarily in nuclei of cells although it emanated from all the tumor tissues (Figure 4). Generally, doxorubicin distributed around tumor blood vessels and decreased with distance from the blood vessels (Figure 5). The fluorescence intensity of doxorubicin also decayed with distance from the blood vessels. It was noted that even in the transcatheter-treated groups many regions of tumor cells were not exposed to detectable concentrations of doxorubicin. These tumor cells were mainly located in avascular and adjacent regions. In addition, there were a few CD31-positive microvessels without surrounding detectable doxorubicin.

**Figure 6:**
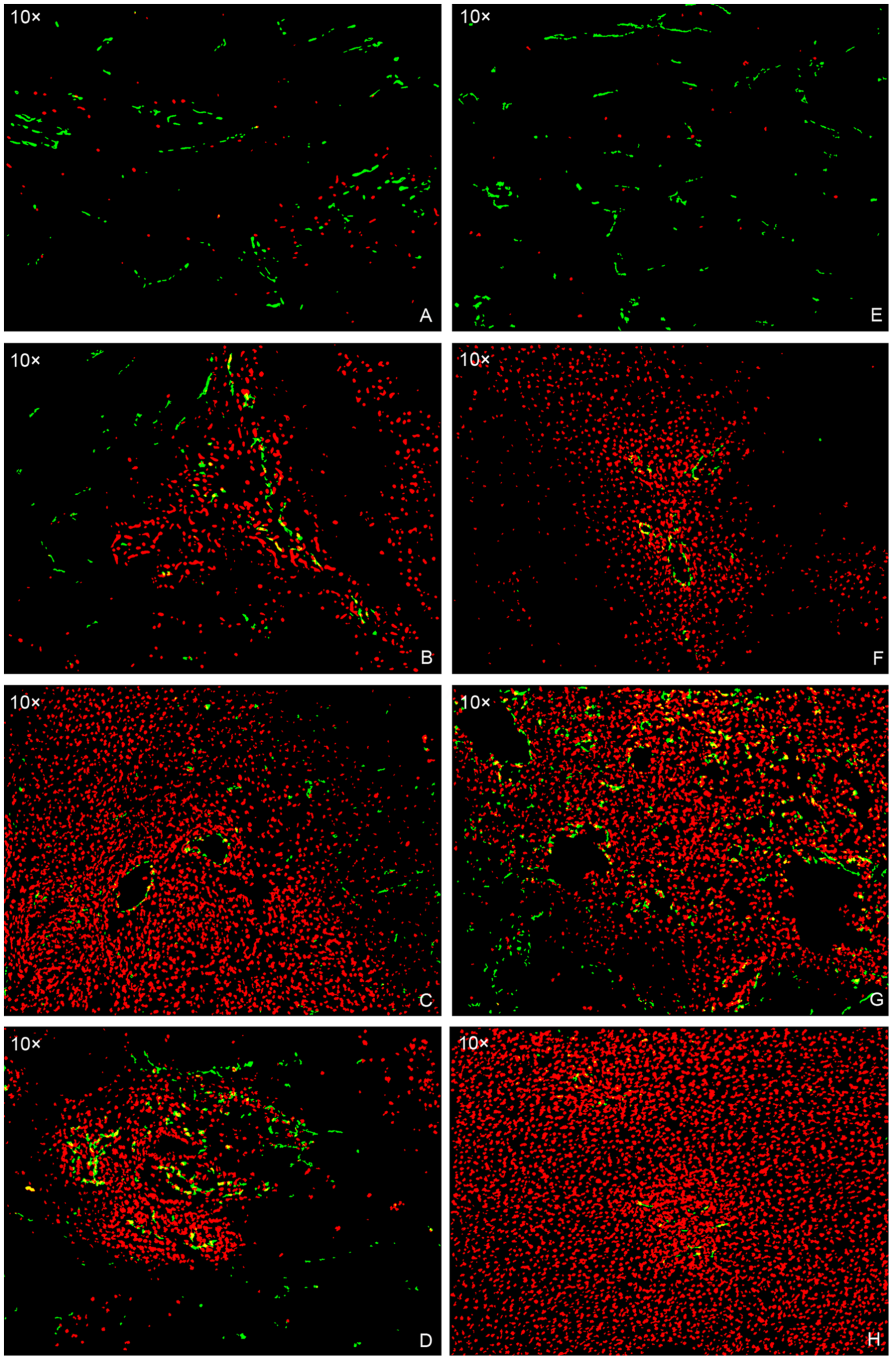
Composite images of doxorubicin and blood vessels. Images shows the difference in doxorubicin penetration between group 1 (A, E), 2 (B, F), 3 (C, G) and 4 (D, H) tumors at 10-minute (A–D) and 4-hour (E–H) time point.

The Table 1 and Figure 6 summarize the doxorubicin penetration distance in four groups based on time of sacrifice. Tumors in the group 2, 3 and 4 had a significant increase in doxorubicin penetration compared with the group 1 tumors at 10 minutes (*P* = 0.032, 0.001 and 0.046, respectively), 4 hours (*P* = 0.046, *P*<0.001 and *P*<0.001, respectively) and in total (*P* = 0.09, *P*<0.001 and *P*<0.001, respectively). Among the three groups of transcatheter therapies, group 3 tumors showed the greatest doxorubicin penetration distance, with significant difference compared with the group 2 and 4 (*P* = 0.010 and 0.007, respectively), and no significant difference was found between group 2 and 4 tumors (*P* = 0.846) at 10 minutes. In contrast, at 4 hours and in total, both group 3 and 4 tumors had a significant increase in drug penetration compared with group 2 (*P* = 0.004 and 0.001, at 4 hours; *P*<0.001 and *P* = 0.023, in total, respectively), and no significant difference was noted between group 3 and 4 tumors (*P* = 0.454, at 4 hours; *P* = 0.138, in total, respectively).

The count of doxorubicin-specific fluorescence spot among the four groups showed a trend of change similar to the trend seen in doxorubicin penetration distance (Table 1). The counts of doxorubicin fluorescence in group 2, 3 and 4 were significantly greater than that in group 1 (*P*<0.05). Among the three groups of transcatheter therapies, there was an increase in drug fluorescent count between group 2 and 3 and between 2 and 4, although the latter was not statistically significant at 10 minutes (*P* = 0.568). No significant difference was found between group 3 and 4 in drug fluorescent count at 4 hours (*P* = 0.170) and in total (*P* = 0.286), although the difference was significant at 10 minutes (*P* = 0.001).

## Discussion

It is well known that to be most effective chemotherapeutic agents must penetrate deep to reach all cancer cells at a therapeutically effective concentration. The aims of hepatic artery chemotherapeutic infusion, with or without subsequent embolization, are to maximize the cytotoxic effects of chemotherapeutic agents and minimize systemic toxicity. Although previous studies have suggested that the transcatheter intraarterial techniques increase intratumoral drug concentration [11], [12] and prolong drug contact time with tumor cells [23], which may partly account for the improved chemotherapeutic efficacy, the role of these techniques on drug distribution remains largely undefined. The data here suggest that transcatheter intraarterial techniques improve the distribution of doxorubicin in liver cancer.

This study demonstrates that transcatheter intraarterial techniques contribute to the penetration of doxorubicin. It is widely believed that the delivery of chemotherapeutic agents to cancer cells classically involves three processes: transport via blood vessels, transport from blood vessels into tumor interstitial space, and transport through interstitial space [24]. These processes are determined by multiple factors including the physicochemical properties of a drug and the biologic properties of a tumor. Recent studies have shown that the transvascular drug transport is mainly affected by microvascular pressure and blood flow in tumor, and that the transport through interstitium of low-molecular-weight drug like doxorubicin is mainly affected by diffusion which depends on the diffusivity and concentration gradient of drug [24], [25]. As shown in our results, the penetration distance of doxorubicin fluorescence was significantly greater in the three groups of transcatheter therapies than that in vein doxorubicin injection group. This finding likely is attributed to the improved drug transport processes generated by transcatheter intraarterial delivery. Firstly, the drug infusion via an intraarterially inserted catheter can directly deliver drug into the tumor-feeding arteries, facilitating the transport via blood vessels. Secondly, the infusion theoretically increases blood flow in tumor tissues, which may improve the transvascular drug transport. Finally, the intraarterial delivery of highly concentrated doses of chemotherapy increases the concentration gradient, which can enhance the drug transport through tumor interstitium.

In addition, our study suggested that transcatheter intraarterial embolization after intraarterial doxorubicin infusion helps further drug penetration. In an earlier review article, Geschwind [26] speculated that particle embolization of tumor-feeding vessels could allow drug to penetrate in tumor with greater ease. Our results showed that both group 3 and group 4 tumors had greater mean doxorubicin penetration distance compared with group 2, which support the hypothesis. It has been shown that a greater microvascular pressure and/or a lower interstitial fluid pressure can increase transvascular fluid filtration, and in turn, lead to an enhancement of transvascular drug transport to tumor [24]. We believe that the significant increase in doxorubicin penetration distance in group 3 and 4 tumors probably be the result of the increased pressure difference generated by subsequent embolization of tumor-feeding vessels.

An interesting finding was that doxorubicin penetration distance in group 4 tumors was significantly smaller than that in group 3 at 10 minutes but was slightly larger than group 3 at 4 hours. This may be due to the different time intervals between drug delivery and necropsy. Previous experimental studies have shown that Lipiodol, as a carrier of anticancer drugs, can increase intratumoral drug concentration [27], but that the material mainly retained in microarterioles and venules after intraarterial infusion as a result of its liposolubility [28]. Lewis and colleagues [20] recently performed doxorubicin release experiment in vitro using a T-cell apparatus and found that the drug eluted from doxorubicin/Lipiodol mixture in less than 4 hours, with a half-life of 1 hour. In this study, extracting tumor tissue 10 minutes after treatment will inevitably underestimate the drug penetration in tumors receiving Lipiodol. In contrast, the 4-hour time interval allows doxorubicin to elute from Lipiodol emulsion, leading to the increase in drug penetration.

By using fluorescence microscopy, we also showed a significantly increased count of doxorubicin fluorescent spot in three groups of transcatheter therapies compared with vein injection group. It is analogous to the findings observed with high performance liquid chromatography in earlier studies [11], [12], [29], which confirm that transcatheter intraarterial techniques improve drug concentration in liver cancer. Among the three transcatheter intraarterial therapy groups, the change trend of doxorubicin fluorescence count was similar to that of doxorubicin penetration distance, which likely is also explained by the embolization-related drug transportation and Lipiodol-caused drug retention.

On the other hand, our study showed that tumor cells in avascular and adjacent regions of tumors receiving transcatheter treatment were not exposed to detectable concentrations of doxorubicin. It is similar to the findings observed in recent studies. Namur and colleagues performed liver transplantation following TACE in patients with hepatocellular carcinoma and found that the doxorubicin concentration in tumor tissue decreased with the distance to the occluded vessels and the drug penetration was associated with tumor necrosis [15]. These results suggest that the effect of transcatheter intraarterial techniques currently used on drug distribution could be somewhat limited. Transcatheter intraarterial techniques, because they depend on the existing vasculature, may improve the drug delivery to vascular regions of tumor but fail to improve the delivery to avascular regions. In the light of these findings, it is clear that additional methods for modifying drug distribution should be introduced in transcatheter intraarterial therapies to achieve better chemotherapeutic efficacy.

This study had several limitations. First, we used an overdose of doxorubicin for facilitating the detection of the drug-specific inflorescence, especially in tumor receiving doxorubicin intravenous injection, which could overestimate the effect of transcatheter intraarterial techniques on drug penetration. The distribution of doxorubicin given in a routine dose via transcatheter intraarterial route needs to be further investigated. Second, we selected 10 minutes and 4 hours after doxorubicin administration as the sacrifice time points to ensure effective drug penetration [19], [20]. Given the fact that drug delivery in tumor is a dynamic process, it is possible that more time points of measurements might provide additional information about doxorubicin penetration in tumor. Finally, we used a sample of the tumor to assess doxorubicin distribution throughout the tumor. The penetration length in two-dimensional images could overestimate the real distance of a doxorubicin fluorescent spot to its nearest vessel since the nearest vessel might be out of the section. Future studies using immunofluorescence technique should aim to quantify staining throughout the entire tumor.

In summary, this study provides evidence that hepatic artery chemotherapeutic infusion, especially when combined with embolization, improves drug penetration as well as drug concentration in liver cancer. This could, at least in part, account for the mechanism by which transcatheter intraarterial techniques are effective therapies for liver cancer. On the other hand, our results suggest that the effect of these techniques on drug distribution is somewhat limited in spite of the overdose of doxorubicin. Further studies are necessary to develop additional strategies for improve the distribution of doxorubicin.
